# Supplementary material for: A Likelihood Approach for Real-Time Calibration of Stochastic Compartmental Epidemic Models
Source: PLoS Comput Biol. 2017 Jan 17;13(1):e1005257. doi: 10.1371/journal.pcbi.1005257 (PMC5240920; doi:10.1371/journal.pcbi.1005257)
Supplement: S1 File — (TAR.GZ) [file pcbi.1005257.s014.tar.gz › HSPH_Online-SI-Revision/PF08/n100-mild/n100-mild_PF2_table-4.pdf]

1	newly 3 weeks spec	3503.	4313.37	{3714., 4938.}	23.6041	0.0898486	newly 3 weeks cumul	8908.	10 188.3	{8879., 11 677.}	15.6642	0.0619541	newly 5 weeks spec	3621.	5947.94	{5171., 6920.}	64.2624	0.213052	newly 5 weeks cumul	16 248.	21 359.4	{18 568., 24 489.}	31.5363	0.116761	newly 56 weeks cumul	29 432.	77 763.6	{73 969., 83 863.}	164.214	0.421587
2	newly 3 weeks spec	4175.	4920.43	{4296., 5485.}	19.0469	0.0746107	newly 3 weeks cumul	10 673.	12 214.7	{10 566., 13 591.}	15.8399	0.0629904	newly 5 weeks spec	4247.	6065.72	{5359., 6750.}	42.8495	0.152899	newly 5 weeks cumul	19 311.	23 897.1	{21 015., 26 477.}	24.1968	0.092553	newly 55 weeks cumul	34 724.	74 400.6	{71 614., 78 488.}	114.263	0.330303
3	newly 3 weeks spec	4167.	4212.52	{3370., 5020.}	12.4324	0.0539915	newly 3 weeks cumul	10 718.	10 041.1	{8315., 11 708.}	10.851	0.0505617	newly 5 weeks spec	4114.	5727.63	{4613., 6912.}	39.3075	0.139023	newly 5 weeks cumul	19 213.	20 823.7	{16 782., 24 575.}	13.7793	0.0558797	newly 58 weeks cumul	31 221.	72 167.3	{64 850., 81 902.}	131.15	0.362209
4	newly 3 weeks spec	8072.	8037.8	{7286., 8571.}	4.42939	0.0195255	newly 3 weeks cumul	18 303.	17 288.2	{15 980., 18 359.}	5.9646	0.0271812	newly 5 weeks spec	6729.	10 247.2	{9797., 10 873.}	52.2846	0.182251	newly 5 weeks cumul	33 292.	37 253.2	{34 533., 38 934.}	11.994	0.0488302	newly 62 weeks cumul	45 410.	84 330.5	{81 275., 91 653.}	85.7091	0.268353
5	newly 3 weeks spec	3934.	5408.38	{4792., 6032.}	37.4779	0.13634	newly 3 weeks cumul	10 428.	12 854.5	{11 475., 14 299.}	23.2885	0.0893704	newly 5 weeks spec	3936.	6902.69	{6231., 7596.}	75.3732	0.242586	newly 5 weeks cumul	18 434.	26 114.4	{23 293., 28 806.}	41.6641	0.149778	newly 58 weeks cumul	32 210.	70 760.3	{66 773., 78 895.}	119.684	0.340967
6	newly 3 weeks spec	4474.	5306.48	{4600., 5767.}	19.2284	0.0753509	newly 3 weeks cumul	11 885.	13 001.1	{11 357., 14 147.}	11.2548	0.046117	newly 5 weeks spec	4394.	6620.66	{5646., 7218.}	50.675	0.176701	newly 5 weeks cumul	20 970.	25 763.7	{22 426., 27 969.}	22.9949	0.0886519	newly 56 weeks cumul	33 539.	77 816.1	{73 908., 80 677.}	132.017	0.365086
7	newly 3 weeks spec	5537.	4890.33	{3949., 5464.}	12.3791	0.0609269	newly 3 weeks cumul	11 792.	10 109.1	{8439., 11 264.}	14.6346	0.0717176	newly 5 weeks spec	6137.	8273.08	{6632., 9067.}	36.042	0.132164	newly 5 weeks cumul	24 548.	25 099.1	{20 512., 27 637.}	9.52892	0.0422286	newly 61 weeks cumul	35 993.	88 945.6	{86 755., 90 851.}	147.119	0.392323
8	newly 3 weeks spec	3810.	5267.89	{4621., 5681.}	38.2648	0.139202	newly 3 weeks cumul	10 632.	13 089.7	{11 396., 13 963.}	23.3272	0.0897894	newly 5 weeks spec	3274.	6379.63	{5856., 6692.}	94.8574	0.288703	newly 5 weeks cumul	17 804.	25 441.2	{22 565., 26 799.}	42.8959	0.153734	newly 55 weeks cumul	26 742.	74 976.9	{73 799., 75 511.}	180.371	0.447572
9	newly 3 weeks spec	6820.	7696.44	{7190., 8139.}	13.4654	0.0545201	newly 3 weeks cumul	16 298.	17 101.1	{16 036., 18 089.}	6.36477	0.0267304	newly 5 weeks spec	6191.	9769.5	{9301., 10 143.}	57.8016	0.197695	newly 5 weeks cumul	29 431.	36 056.1	{34 142., 37 842.}	22.5774	0.0878968	newly 60 weeks cumul	43 358.	88 074.	{86 644., 89 292.}	103.132	0.307566
10	newly 3 weeks spec	3895.	4307.66	{3955., 4632.}	10.8334	0.0439551	newly 3 weeks cumul	10 575.	11 133.7	{10 250., 11 898.}	6.65296	0.0277767	newly 5 weeks spec	3765.	5091.49	{4708., 5449.}	35.2321	0.130214	newly 5 weeks cumul	18 373.	21 010.7	{19 464., 22 342.}	14.3566	0.0575796	newly 54 weeks cumul	31 484.	65 342.6	{62 546., 69 954.}	107.542	0.316549
11	newly 3 weeks spec	5349.	3852.74	{3490., 4244.}	27.9727	0.143757	newly 3 weeks cumul	11 903.	8695.28	{7880., 9619.}	26.9488	0.137574	newly 5 weeks spec	6127.	5929.8	{5361., 6421.}	5.42974	0.0245008	newly 5 weeks cumul	24 206.	19 560.8	{17 566., 21 451.}	19.1901	0.0936212	newly 58 weeks cumul	41 009.	79 287.2	{78 323., 81 119.}	93.3409	0.286281
12	newly 3 weeks spec	4227.	4842.55	{4326., 5373.}	14.8429	0.0588453	newly 3 weeks cumul	11 278.	12 339.8	{11 042., 13 648.}	10.5301	0.0426976	newly 5 weeks spec	4123.	5706.88	{5095., 6215.}	38.4157	0.140159	newly 5 weeks cumul	19 649.	23 434.1	{21 089., 25 838.}	19.2636	0.075279	newly 54 weeks cumul	37 494.	70 093.3	{67 069., 74 210.}	86.9454	0.271461
13	newly 3 weeks spec	5374.	5570.48	{5018., 5954.}	7.15296	0.0285987	newly 3 weeks cumul	12 699.	12 453.8	{11 326., 13 164.}	7.41515	0.0315162	newly 5 weeks spec	5146.	7867.21	{7195., 8462.}	52.8801	0.182731	newly 5 weeks cumul	23 362.	27 279.1	{24 791., 28 914.}	16.8078	0.065406	newly 59 weeks cumul	36 672.	77 221.8	{71 115., 85 110.}	110.574	0.321923
14	newly 3 weeks spec	7541.	9576.18	{9372., 9787.}	26.9882	0.103662	newly 3 weeks cumul	19 010.	21 788.4	{21 416., 22 197.}	14.6154	0.0591354	newly 5 weeks spec	5669.	10 619.5	{10 405., 10 826.}	87.3262	0.272485	newly 5 weeks cumul	32 236.	43 138.5	{42 521., 43 724.}	33.8208	0.126448	newly 61 weeks cumul	43 111.	89 753.6	{88 988., 90 855.}	108.192	0.318388
15	newly 3 weeks spec	6533.	4998.34	{4721., 5200.}	23.4909	0.116665	newly 3 weeks cumul	14 286.	11 220.2	{10 625., 11 671.}	21.4602	0.105248	newly 5 weeks spec	6860.	7292.04	{6967., 7549.}	6.5758	0.0275303	newly 5 weeks cumul	28 288.	24 802.5	{23 657., 25 655.}	12.3215	0.0573869	newly 58 weeks cumul	44 155.	81 688.6	{81 285., 82 061.}	85.0042	0.267178
16	newly 3 weeks spec	2942.	3609.99	{3275., 3958.}	22.7053	0.0876839	newly 3 weeks cumul	7823.	9045.67	{8239., 9731.}	15.6292	0.062206	newly 5 weeks spec	2786.	4666.93	{4220., 5285.}	67.5136	0.22256	newly 5 weeks cumul	13 642.	17 896.8	{16 256., 19 387.}	31.1888	0.116834	newly 54 weeks cumul	27 181.	69 851.9	{65 888., 76 486.}	156.988	0.409241
17	newly 3 weeks spec	6206.	6593.65	{5263., 7394.}	12.0411	0.0514184	newly 3 weeks cumul	13 556.	14 062.	{11 565., 15 448.}	9.54065	0.0414392	newly 5 weeks spec	6327.	9484.12	{8021., 10 208.}	50.0901	0.174297	newly 5 weeks cumul	26 549.	31 941.4	{26 521., 34 918.}	22.1772	0.086482	newly 60 weeks cumul	43 173.	89 524.6	{87 303., 91 336.}	107.362	0.316458
18	newly 3 weeks spec	7370.	5592.28	{3196., 7941.}	29.2046	0.167939	newly 3 weeks cumul	13 588.	10 170.4	{6408., 14 149.}	27.6002	0.156678	newly 5 weeks spec	8646.	10 690.6	{6472., 14 204.}	32.522	0.118692	newly 5 weeks cumul	31 571.	29 403.4	{17 860., 40 318.}	27.5675	0.132304	newly 64 weeks cumul	43 150.	91 601.1	{84 422., 93 545.}	112.285	0.326533
19	newly 3 weeks spec	4209.	3939.2	{3337., 4407.}	8.33167	0.0393031	newly 3 weeks cumul	10 513.	9633.91	{8130., 10 781.}	9.27281	0.0443439	newly 5 weeks spec	4445.	5198.31	{4588., 5576.}	17.4873	0.0689858	newly 5 weeks cumul	19 470.	19 464.4	{16 809., 21 486.}	8.31448	0.0368606	newly 55 weeks cumul	33 382.	73 068.4	{69 663., 76 457.}	118.886	0.339993
20	newly 3 weeks spec	5435.	3757.	{1640., 7031.}	43.8638	0.269231	newly 3 weeks cumul	9479.	6833.46	{3319., 12 126.}	39.9582	0.234664	newly 5 weeks spec	7800.	8096.64	{3585., 13 935.}	39.7149	0.176762	newly 5 weeks cumul	25 122.	20 933.1	{9619., 37 142.}	40.959	0.218809	newly 64 weeks cumul	39 842.	86 355.	{73 637., 94 134.}	116.744	0.333959
21	newly 3 weeks spec	6939.	6872.25	{4831., 8865.}	19.6972	0.0884432	newly 3 weeks cumul	13 970.	13 441.4	{9523., 16 921.}	17.254	0.0795914	newly 5 weeks spec	7245.	10 760.7	{8188., 12 784.}	49.0552	0.167	newly 5 weeks cumul	29 481.	33 611.5	{24 281., 41 659.}	21.6533	0.0851728	newly 64 weeks cumul	42 520.	85 988.3	{81 045., 92 428.}	102.23	0.303008
22	newly 3 weeks spec	3601.	4412.42	{4044., 4741.}	22.5332	0.0874478	newly 3 weeks cumul	9861.	11 285.9	{10 552., 12 061.}	14.5879	0.0585341	newly 5 weeks spec	3826.	5286.22	{4871., 5639.}	38.1657	0.139742	newly 5 weeks cumul	17 518.	21 516.2	{20 063., 23 003.}	22.8235	0.088618	newly 54 weeks cumul	36 374.	67 627.1	{63 432., 70 482.}	85.9216	0.268938
23	newly 3 weeks spec	5953.	4153.21	{3833., 4527.}	30.2333	0.157419	newly 3 weeks cumul	13 022.	9179.1	{8468., 9876.}	29.5108	0.152777	newly 5 weeks spec	6752.	6559.17	{5993., 7201.}	5.7386	0.0255421	newly 5 weeks cumul	26 990.	21 151.2	{19 541., 23 028.}	21.6331	0.10673	newly 59 weeks cumul	42 711.	80 977.6	{70 492., 83 357.}	89.5941	0.277186
24	newly 3 weeks spec	7664.	5445.96	{4443., 6950.}	28.941	0.153393	newly 3 weeks cumul	15 273.	11 162.9	{9180., 14 080.}	26.9108	0.140409	newly 5 weeks spec	6606.	8973.96	{7687., 10 471.}	35.8456	0.130348	newly 5 weeks cumul	30 012.	27 582.2	{23 221., 33 782.}	12.1596	0.0569125	newly 63 weeks cumul	39 935.	88 342.5	{83 918., 92 367.}	121.216	0.34412
25	newly 3 weeks spec	4080.	3258.69	{3011., 3508.}	20.1301	0.0983312	newly 3 weeks cumul	9690.	8070.88	{7472., 8566.}	16.7092	0.0800242	newly 5 weeks spec	4627.	4336.02	{4056., 4605.}	6.49924	0.0297207	newly 5 weeks cumul	18 986.	16 222.9	{15 200., 17 175.}	14.5532	0.068898	newly 54 weeks cumul	37 102.	72 242.6	{71 344., 73 118.}	94.7134	0.289361
26	newly 3 weeks spec	5222.	4910.42	{4548., 5231.}	6.59747	0.0300493	newly 3 weeks cumul	12 723.	11 690.7	{10 913., 12 465.}	8.32013	0.038182	newly 5 weeks spec	5352.	6512.22	{6040., 6903.}	21.6783	0.084606	newly 5 weeks cumul	23 692.	24 026.4	{22 391., 25 572.}	4.30774	0.0184758	newly 56 weeks cumul	38 625.	79 543.7	{76 096., 82 677.}	105.938	0.313474
27	newly 3 weeks spec	4332.	3426.18	{3171., 3655.}	20.91	0.102525	newly 3 weeks cumul	10 828.	8492.55	{7882., 8952.}	21.5686	0.106107	newly 5 weeks spec	4581.	4548.72	{4210., 4785.}	3.6778	0.0161514	newly 5 weeks cumul	20 103.	17 068.8	{15 895., 18 023.}	15.0935	0.0715596	newly 54 weeks cumul	36 608.	73 101.3	{72 344., 73 898.}	99.6867	0.300335
28	newly 3 weeks spec	3391.	4098.77	{3471., 4678.}	21.3444	0.0813824	newly 3 weeks cumul	9081.	10 142.2	{8574., 11 568.}	13.7628	0.0547553	newly 5 weeks spec	3182.	5308.66	{4496., 5898.}	66.8341	0.219542	newly 5 weeks cumul	15 833.	20 234.4	{17 015., 22 841.}	27.9159	0.104359	newly 55 weeks cumul	31 926.	71 705.8	{67 598., 73 923.}	124.6	0.350916
29	newly 3 weeks spec	3449.	4049.57	{3415., 4588.}	19.0348	0.074382	newly 3 weeks cumul	8407.	9464.47	{8139., 10 611.}	14.74	0.0590574	newly 5 weeks spec	3362.	5798.43	{4803., 6476.}	72.4697	0.233994	newly 5 weeks cumul	15 415.	20 240.9	{17 215., 22 768.}	31.5064	0.116627	newly 57 weeks cumul	26 694.	79 173.2	{75 725., 82 487.}	196.595	0.471612
30	newly 3 weeks spec	6917.	7270.76	{5023., 7999.}	14.2805	0.0629904	newly 3 weeks cumul	15 845.	15 980.7	{11 162., 17 588.}	10.7115	0.0495339	newly 5 weeks spec	6557.	9607.42	{7382., 10 324.}	46.5216	0.163078	newly 5 weeks cumul	29 817.	34 452.8	{24 832., 37 758.}	21.0345	0.0847577	newly 59 weeks cumul	44 183.	87 822.3	{73 311., 90 543.}	98.7694	0.297305
31	newly 3 weeks spec	4050.	4425.45	{4080., 4751.}	9.54593	0.0389953	newly 3 weeks cumul	9667.	10 461.7	{9689., 11 115.}	8.57195	0.0352179	newly 5 weeks spec	4228.	6099.52	{5703., 6520.}	44.2649	0.158545	newly 5 weeks cumul	18 314.	21 917.7	{20 349., 23 430.}	19.6772	0.0773973	newly 56 weeks cumul	33 046.	79 278.2	{75 950., 82 419.}	139.902	0.379777
32	newly 3 weeks spec	3828.	4600.91	{4070., 5298.}	20.191	0.0781348	newly 3 weeks cumul	10 480.	11 781.8	{10 450., 13 466.}	12.7771	0.0508657	newly 5 weeks spec	3614.	5452.37	{4902., 5967.}	50.868	0.177322	newly 5 weeks cumul	17 929.	22 370.1	{20 022., 25 198.}	24.7707	0.0946694	newly 54 weeks cumul	35 777.	68 782.2	{65 774., 71 120.}	92.2526	0.283532
33	newly 3 weeks spec	6228.	1463.83	{458., 2729.}	76.496	0.730701	newly 3 weeks cumul	10 354.	2854.54	{1139., 5005.}	72.4306	0.637393	newly 5 weeks spec	7272.	3436.59	{763., 7036.}	54.8731	0.472766	newly 5 weeks cumul	26 028.	8626.27	{2603., 16 625.}	67.429	0.59329	newly 64 weeks cumul	32 752.	78 647.6	{57 441., 93 500.}	140.131	0.373186
34	newly 3 weeks spec	7515.	7054.35	{6484., 7467.}	6.37911	0.0291243	newly 3 weeks cumul	17 041.	15 539.8	{14 341., 16 441.}	8.8415	0.0408332	newly 5 weeks spec	6043.	9402.86	{9055., 9735.}	55.5992	0.191855	newly 5 weeks cumul	30 687.	33 563.6	{31 520., 35 200.}	9.42295	0.0387595	newly 60 weeks cumul	40 483.	87 833.7	{87 184., 88 474.}	116.964	0.336381
35	newly 3 weeks spec	11 144.	673.64	{245., 1171.}	93.9551	1.31011	newly 3 weeks cumul	14 898.	1386.06	{556., 2234.}	90.6963	1.10022	newly 5 weeks spec	7436.	1557.27	{366., 2994.}	79.0577	0.820002	newly 5 weeks cumul	37 536.	3976.63	{1252., 7211.}	89.4058	1.07974	newly 64 weeks cumul	40 652.	74 136.	{54 235., 90 369.}	82.3675	0.252667
36	newly 3 weeks spec	4855.	5084.21	{2734., 7942.}	34.2799	0.146113	newly 3 weeks cumul	9201.	9357.66	{5272., 13 886.}	29.4125	0.128225	newly 5 weeks spec	5772.	9885.93	{6008., 14 229.}	73.3096	0.220156	newly 5 weeks cumul	20 947.	27 027.4	{15 453., 40 056.}	41.0258	0.144964	newly 64 weeks cumul	32 008.	91 470.2	{85 495., 93 952.}	185.773	0.455501
37	newly 3 weeks spec	5673.	5065.17	{4687., 5409.}	10.7144	0.0499546	newly 3 weeks cumul	13 815.	11 977.7	{11 014., 12 730.}	13.2992	0.0626738	newly 5 weeks spec	5464.	6762.81	{6287., 7151.}	23.7703	0.0921128	newly 5 weeks cumul	25 233.	24 800.	{23 027., 26 319.}	4.09733	0.0182646	newly 57 weeks cumul	39 323.	78 157.7	{76 845., 78 984.}	98.7581	0.298291
38	newly 3 weeks spec	6180.	1893.03	{776., 3040.}	69.3684	0.569392	newly 3 weeks cumul	10 916.	3772.25	{1749., 5806.}	65.4429	0.502808	newly 5 weeks spec	7800.	4155.75	{1203., 7264.}	48.3073	0.357609	newly 5 weeks cumul	26 749.	10 845.1	{3911., 18 007.}	59.921	0.454932	newly 64 weeks cumul	37 595.	77 672.5	{58 552., 92 865.}	106.603	0.310217
39	newly 3 weeks spec	5821.	8144.17	{7479., 9158.}	39.9102	0.144554	newly 3 weeks cumul	14 912.	18 194.8	{16 673., 20 211.}	22.044	0.0852643	newly 5 weeks spec	4560.	9977.09	{9350., 10 741.}	118.796	0.339507	newly 5 weeks cumul	25 319.	37 745.7	{35 032., 41 698.}	49.0805	0.172541	newly 61 weeks cumul	34 866.	84 076.7	{79 323., 91 714.}	141.142	0.381838
40	newly 3 weeks spec	8986.	9972.04	{7096., 11 093.}	18.0525	0.0750264	newly 3 weeks cumul	18 610.	19 602.5	{14 386., 21 708.}	12.9703	0.0561707	newly 5 weeks spec	6981.	12 969.8	{10 775., 13 708.}	85.7866	0.26753	newly 5 weeks cumul	34 664.	45 151.1	{34 489., 49 103.}	30.4652	0.112776	newly 63 weeks cumul	43 420.	93 514.9	{93 036., 93 956.}	115.373	0.333188
41	newly 3 weeks spec	8035.	4996.61	{2822., 8682.}	41.1177	0.254872	newly 3 weeks cumul	14 755.	9410.5	{5934., 15 238.}	38.3988	0.231433	newly 5 weeks spec	8819.	9280.07	{5474., 14 167.}	26.0459	0.108754	newly 5 weeks cumul	33 711.	26 085.5	{15 609., 41 657.}	34.1036	0.182682	newly 64 weeks cumul	47 457.	89 811.3	{80 518., 93 707.}	89.2478	0.275655
42	newly 3 weeks spec	6821.	6332.86	{5452., 7030.}	8.39173	0.0399349	newly 3 weeks cumul	14 678.	13 115.2	{11 652., 14 437.}	10.7526	0.0513068	newly 5 weeks spec	6589.	9723.43	{8591., 10 405.}	47.5706	0.167451	newly 5 weeks cumul	28 856.	31 221.4	{27 323., 33 870.}	11.2267	0.0467125	newly 61 weeks cumul	41 167.	90 892.1	{88 573., 92 315.}	120.789	0.343892
43	newly 3 weeks spec	6531.	4765.23	{2768., 8201.}	38.4332	0.213097	newly 3 weeks cumul	12 527.	8942.86	{5649., 14 697.}	36.1086	0.201035	newly 5 weeks spec	8332.	9133.58	{5770., 14 015.}	26.837	0.109197	newly 5 weeks cumul	29 200.	25 237.1	{15 394., 40 671.}	30.605	0.150924	newly 64 weeks cumul	45 957.	91 377.6	{85 863., 93 844.}	98.8328	0.298163
44	newly 3 weeks spec	8080.	5069.19	{4035., 5615.}	37.2625	0.205056	newly 3 weeks cumul	15 523.	9926.9	{8081., 10 944.}	36.0504	0.196468	newly 5 weeks spec	8641.	9248.23	{7809., 10 065.}	10.2218	0.0426232	newly 5 weeks cumul	34 157.	26 517.5	{22 325., 28 777.}	22.3659	0.111821	newly 63 weeks cumul	46 278.	88 289.3	{82 913., 93 814.}	90.7802	0.279846
45	newly 3 weeks spec	4973.	5175.05	{4438., 5773.}	9.96964	0.0421318	newly 3 weeks cumul	11 874.	12 022.4	{10 386., 13 440.}	8.29687	0.0358673	newly 5 weeks spec	4999.	6978.42	{6036., 7752.}	39.5963	0.142812	newly 5 weeks cumul	22 191.	25 264.1	{21 835., 28 328.}	15.0371	0.0595558	newly 59 weeks cumul	37 756.	72 634.6	{65 325., 83 692.}	92.379	0.282325
46	newly 3 weeks spec	6137.	5562.79	{4619., 6130.}	9.88708	0.0484672	newly 3 weeks cumul	13 041.	11 540.4	{9906., 12 802.}	11.6802	0.0565679	newly 5 weeks spec	7235.	8984.99	{7219., 9662.}	26.7589	0.10328	newly 5 weeks cumul	27 607.	28 037.	{23 132., 30 526.}	8.00739	0.0362491	newly 60 weeks cumul	46 416.	89 366.6	{87 173., 91 539.}	92.534	0.28348
47	newly 3 weeks spec	7497.	6154.27	{5661., 6562.}	17.9102	0.0863907	newly 3 weeks cumul	15 877.	13 129.2	{12 147., 13 845.}	17.307	0.0831016	newly 5 weeks spec	7293.	9121.68	{8618., 9640.}	25.0745	0.0966621	newly 5 weeks cumul	31 506.	30 194.2	{28 231., 31 809.}	4.71472	0.0213452	newly 60 weeks cumul	45 078.	88 918.7	{87 765., 90 002.}	97.2552	0.294999
48	newly 3 weeks spec	5435.	3171.11	{3019., 3354.}	41.6539	0.234322	newly 3 weeks cumul	12 559.	7687.83	{7349., 8050.}	38.7863	0.213392	newly 5 weeks spec	5927.	4439.83	{4201., 4635.}	25.0914	0.125779	newly 5 weeks cumul	24 449.	15 957.1	{15 243., 16 688.}	34.7331	0.185552	newly 55 weeks cumul	39 759.	75 361.6	{74 666., 75 929.}	89.546	0.277707
49	newly 3 weeks spec	5594.	4492.78	{3881., 5149.}	19.9546	0.0995841	newly 3 weeks cumul	12 132.	9785.55	{8629., 11 201.}	19.4247	0.0962752	newly 5 weeks spec	4978.	7082.18	{6199., 8205.}	42.4182	0.150792	newly 5 weeks cumul	22 959.	22 771.	{20 078., 25 939.}	8.94904	0.0392939	newly 61 weeks cumul	32 589.	82 154.1	{75 013., 87 520.}	152.092	0.400173
50	newly 3 weeks spec	6652.	7135.62	{6655., 7554.}	8.32201	0.0346372	newly 3 weeks cumul	14 670.	14 652.4	{13 920., 15 497.}	4.00579	0.0175791	newly 5 weeks spec	6004.	10 462.5	{9661., 10 864.}	74.2587	0.240736	newly 5 weeks cumul	27 698.	34 399.5	{32 422., 36 028.}	24.247	0.0937999	newly 62 weeks cumul	37 321.	90 881.	{82 401., 93 149.}	143.512	0.386138
